# Gemcitabine and docetaxel combination chemotherapy for advanced bone and soft tissue sarcomas: protocol for an open-label, non-randomised, Phase 2 study

**DOI:** 10.1186/s12885-019-5923-7

**Published:** 2019-07-23

**Authors:** Hitomi Hara, Teruya Kawamoto, Naomasa Fukase, Yohei Kawakami, Toshiyuki Takemori, Shuichi Fujiwara, Kazumichi Kitayama, Kotaro Nishida, Ryosuke Kuroda, Toshihiro Akisue

**Affiliations:** 1 Department of Orthopaedic Surgery, Kobe University Graduate School of Medicine, 7-5-1 Kusunoki-cho, Chuo-ku, Kobe, 650-0017 Japan; 2 Division of Orthopaedic Surgery, Kobe University International Clinical Cancer Research Centre, 1-5-1 Minatojimaminami-cho, Chuo-ku, Kobe, 650-0047 Japan; 3 Department of Rehabilitation Science, Kobe University Graduate School of Health Sciences, 7-10-2 Tomogaoka, Suma-ku, Kobe, 654-0142 Japan

**Keywords:** Bone and soft tissue sarcomas, Gemcitabine and docetaxel, Phase 2 study

## Abstract

**Background:**

The prognosis of patients with metastatic or advanced sarcomas is poor and there are few options for treatment. Several studies have shown that gemcitabine and docetaxel (GD) combination chemotherapy has antitumor activity against various subtypes of sarcoma. Recently, some studies have shown a favourable outcome for GD combination chemotherapy for relapsed high-grade osteosarcoma and spindle cell sarcoma of bone. If the effectiveness of GD is proven, this will result in new treatment options for advanced bone and soft tissue sarcomas (STS). The aim of this prospective Phase 2 study is to evaluate the efficacy and toxicity of the GD combination in patients with advanced bone sarcomas and STS.

**Methods:**

This is a Phase 2, single-arm, open-label study to investigate the efficacy and safety of combination chemotherapy with GD for advanced bone sarcomas and STS and will enrol 20 patients. The patients will receive gemcitabine 900 mg/m^2^ on Days 1 and 8, and docetaxel 70 mg/m^2^ on Day 8 in 3-week cycles until disease progression or other evidence of treatment failure. The primary aim of this study is to analyse GD’s effect on progression-free survival (PFS). The secondary objectives are to analyse treatment efficacy and safety in terms of response rate, tumour control rate, overall survival, and adverse event rate. The length of follow-up will be 5 years.

**Discussion:**

This study will evaluate the efficacy and safety of combination therapy with gemcitabine and docetaxel for bone sarcomas and STS. If this combination proves to be acceptable, it could be used for as second, third, or later line therapy for patients with sarcomas (especially bone sarcomas). In the future, the role of various treatments, including GD therapy, will be clarified for specific subtypes of sarcoma.

**Trial registration:**

This study was registered as UMIN000031004 (University Hospital Medical Information Network-Clinical Trial Registry: UMIN-CTR) on 1 March 1 2018 and with the Japan Registry of Clinical Trials (jRCT) as jRCTs051180042 on 30 January 2019. The posted information will be updated as needed to reflect protocol amendments and study progress.

**Electronic supplementary material:**

The online version of this article (10.1186/s12885-019-5923-7) contains supplementary material, which is available to authorized users.

## Background

Sarcomas are a heterogeneous group of rare mesenchymal malignant tumours. Chemotherapy has a proven role in the treatment of primary bone sarcomas, including osteosarcoma and Ewing sarcoma. Small round-cell soft tissue sarcomas (STS) such as rhabdomyosarcoma and Ewing sarcoma, which usually occur in children and young adults, are sensitive to chemotherapy. Osteosarcoma and small round-cell sarcomas are therefore treated with chemotherapy in combination with surgery or radiation therapy. The therapy for high-risk patients with high-grade large non-small round-cell STS, which usually occurs in older adults, is usually surgery and chemotherapy. There is no established chemotherapy for bone sarcomas other than osteosarcoma and Ewing sarcoma.

A combination chemotherapy regimen comprising methotrexate, doxorubicin, and cisplatin (MAP) is used for osteosarcoma, whereas regimens including vincristine, doxorubicin, ifosfamide, and actinomycin D (VAIA) or vincristine, doxorubicin, and cyclophosphamide plus ifosfamide and etoposide (VDC-IE) are used for Ewing sarcoma. Vincristine, doxorubicin, and cyclophosphamide (VAC) is used for rhabdomyosarcoma and doxorubicin plus ifosfamide for non-round cell STS. Because no standard chemotherapy for bone sarcomas other than osteosarcoma and Ewing sarcoma has yet been established, combination chemotherapy based on cisplatin, doxorubicin, and ifosfamide is mainly used; however, the efficacy is still uncertain. Prognosis of patients with advanced soft tissue and bone sarcomas is poor. Adriamycin-based combination chemotherapy results in a higher response rate than other combinations, ranging from 25 to 40%, but median overall survival (OS) is only 8–12 months [1, 2]. Similarly, adult patients with metastatic bone sarcomas have a 5-year overall survival of less than 25% [3, 4].

The anticancer agents gemcitabine and docetaxel are used to treat various malignant tumours, including non-small cell lung cancer and breast cancer. Combination chemotherapy with gemcitabine and docetaxel (GD) has been shown to be effective for metastatic leiomyosarcoma and other STS [5–10]. Recently, GD has been used for recurrent or refractory osteosarcoma and other bone sarcomas because several studies showed that this combination is effective [10–12]. Chemotherapeutic options for advanced sarcomas are limited. Recent options used in patients with advanced STS have included pazopanib, trabectedin, and eribulin. There are few options for second- or third-line therapy for refractory or metastatic bone sarcomas that has previously been treated with standard chemotherapy. If the effectiveness of GD is proven, it has the potential to result in new treatment options for advanced bone sarcomas and STS. Subtypes of sarcoma also respond differently to various chemotherapy drugs and treatment regimens. Future studies should analyse the response pattern of patients with different histologic subtypes of bone and soft tissue sarcoma. The aim of this Phase 2 study is to evaluate the efficacy and toxicity of GD in patients with advanced bone sarcomas and STS.

## Methods

### Study objectives

The primary aim of this study is to analyse the GD therapy effect on progression-free survival (PFS). The secondary objectives are to analyse treatment efficacy and safety by assessing response rate, tumour control rate, overall survival and adverse event rate.

The target sample size is based on expected response rate; however, the primary endpoint is defined as PFS because prolonging time to disease progression is important even if the response rate is less than expected.

### Design

**Fig. 1:**
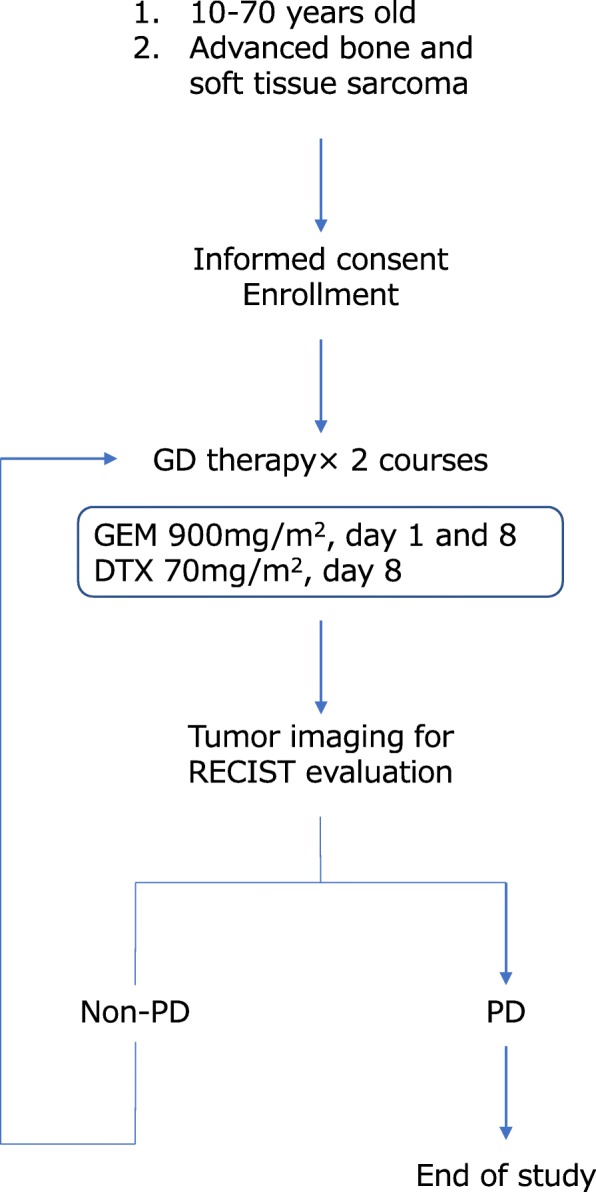
Flowchart of Phase 2 study of gemcitabine and docetaxel combination chemotherapy for sarcomas

This is a Phase 2, single-arm, open-label study to investigate the efficacy and safety of combination chemotherapy with gemcitabine and docetaxel (GD) for advanced bone sarcomas and STS. Patients will receive gemcitabine 900 mg/m^2^ on Days 1 and 8, and docetaxel 70 mg/m^2^ on Day 8, repeated at 21 days intervals until disease progression or other evidence of treatment failure. Patients will undergo computed tomography or magnetic resonance imaging and be evaluated for response after every second cycle using Response Evaluation Criteria in Solid Tumours (RECIST). The total duration of the study will be 5 years; all participants will attend for follow-up every 4–8 weeks after progression or treatment failure. A flowchart of the study design is presented in Fig. 1.

### Patient cohort

The participants in this study will be recruited at Kobe University Hospital.

The inclusion criteria for this study are: (1) histopathologic diagnosis of primary malignant sarcoma of bone and soft tissue in extremity or trunk; (2) extremity or trunk bone sarcoma or STS presenting with advanced recurrence or metastatic disease diagnosed by biopsy as necessary; (3) previous received standard therapy for bone sarcoma or STS, or was unable to receive standard therapy; (4) age 10–70 years at the time of enrolment; (5) measurable lesion(s); (6) performance status (PS) ECOG 0 or 1; (7) primary tumour was in the limbs or trunk; and (8) laboratory data no more than14 days prior to enrolment meeting the following criteria: (i) neutrophil count > 1,500/mm^3^; (ii) haemoglobin > 8.0 g/dL (no blood transfusion within 14 days); (iii) platelet count > 100,000/mm^3^; (iv) total bilirubin < 1.5 mg/dL; (v) aspartate aminotransferase (glutamyl oxaloacetic transaminase) (AST [GOT]) < 100 IU/L; (vi) alanine aminotransferase (glutamyl pyruvic transaminase) (ALT [GPT]) < 100 IU/L; (vii) creatinine < 1.5 mg/dL; (viii) creatinine clearance (eGFR) > 60 mL/min; (ix) normal electrocardiogram no more than 28 days prior to enrolment; (x) no interstitial pneumonia, pulmonary fibrosis, or pulmonary emphysema; and (xi) written informed consent obtained after patients has been given a written explanation of the study protocol. Patients meeting any of the following criteria will be excluded from this study: (1) active double cancers within 5 years (cured intraepithelial carcinoma and intramucosal carcinoma not included); (2) severe infection; (3) significant fever; (4) pregnancy or breastfeeding; (5) severe mental illness; (6) receiving continuous whole-body administration of steroids or other immunosuppressants; (7) unstable angina (within the past 3 months), myocardial infarction; (8) difficult-to-control hypertension; (9) difficult-to-control diabetes; (10) positive hepatitis B antigen; and (11) the final decision to enrol will be up to each patient’s physician.

### Target sample size and rationale

A sample size of 16 will be required for a threshold response rate of 6% (based on the results of previous studies) and an expected response rate of 25%, with a one-sided α of 0.1 and a β of approximately 0.2. To allow for four dropouts, the target sample size of this study has been set to 20. Assuming that four patients will be enrolled per year, the enrolment period has been set to 5 years.

### Study treatment

#### Drugs used

The drugs will be administered in accordance with instructions in the package inserts.

Gemcitabine (Gemzar®, Eli Lilly Oncology, Indianapolis IN, USA), Docetaxel (Taxotere®, Sanofi-Aventis, Paris, France)

The study patients will receive the marketed drugs that are available at the medical institution.

#### Protocol treatment

Protocol treatment should be started within 14 days after enrolment (day of enrolment counted as day zero; thus, up to the same day of the week after the next one is acceptable), and repeated in 3-week cycles until the criteria for treatment withdrawal (below) have been met. The drugs may be administered only after the participant has been confirmed to fulfil the following criteria within 3 days before Day 1 of each cycle: haemoglobin ≥8.0 g/dL, neutrophil count ≥1,000/mm^3^, platelet count ≥50,000/mm^3^, AST (GOT) ≤ 90 IU/L, ALT (GPT) ≤ 125 IU/L for male and ≤ 69 IU/L for female participants, creatinine ≤1.605 mg/dL for male and ≤ 1.185 mg/dL for female participants, grade 0–2 fatigue, grade 0–2 oedema limbs, grade 0–1 diarrhoea, grade 0–1 haematuria, grade 0–1 mucositis oral, grade 0–1 supraventricular tachycardia, grade 0–1 ventricular arrhythmia, grade 0–1 pneumonitis, and grade 0–1 infection. The drugs may be administered only after the participant has been confirmed to fulfil the following criteria within 3 days before Day 8: haemoglobin ≥8.0 g/dL, neutrophil count ≥1,000/mm^3^, platelet count ≥50,000/mm^3^, AST (GOT) ≤ 90 IU/L, ALT (GPT) ≤ 125 IU/L for male and ≤ 69 IU/L for female participants. The drugs should not be administered on Day 8 if the participant has Grade 2 or worse pneumonitis. If the above criteria are not fulfilled, protocol treatment should be withheld until they are met. If initiation of treatment is still considered contraindicated 15 days after Day 1 of that cycle, the patient will be classified as a “protocol treatment withdrawal”.

#### Criteria for dose reduction

Doses will be reduced in participants with any toxicity meeting the criteria listed in Table 1 (adverse events for which a causal relationship to the protocol treatment cannot be ruled out). A 20% reduction in doses of gemcitabine and docetaxel is suggested. Dose modifications are allowed once, the dosage cannot be re-escalated. If toxicity does not abate during the monitoring period, administration of gemcitabine or docetaxel will be interrupted and/or the dose further reduced. The protocol treatment should be permanently discontinued for any haematological or non-haematological toxicity requiring an interruption of ≥14 days.

**Table 1:** Criteria for dose suspension or reduction

Toxicity	Grade	Resumption plan
Neutrophil count (with preventive treatment by G-CSF^a^)	Grade 4 (lasting for 5 days or more)	Preventive treatment by G-CSF.
Neutrophil count (without preventive treatment by G-CSF)	Grade 4 (lasting for 5 days or more)	Reduced dose of 720 mg/m2 of GEM^a^ and 55mg/m2 of DTX^a^ (first occurrence), then 570 mg/m2 of GEM and 44mg/m2 of DTX (second occurrence).
Diarrhea	Grade 3	
Mucositis oral	Grade 3	
Infection	Grade 3	
Pneumonitis	Grade 1	
	Grade 2	Discontinue protocol treatment.
Neuropathy	Grade 2	Reduced dose of 720 mg/m2 of GEM and 55mg/m2 of DTX (first occurrence), then 570 mg/m2 of GEM and 44mg/m2 of DTX (second occurrence).
	Grade 3	Discontinue protocol treatment.
Creatinine	Grade 2-4	
Supraventricular tachycardia	Grade 2 (two times), Grade 3	
Atrial arrhythmia	Grade 2 (two times), Grade 3	
Left ventriclar contractile dysfunction	Grade 2 (two times), Grade 3	
Vertigo/Dizziness	Grade 2-3	
Depressed level of consciousness	Grade 2-3	
Seizure	Grade 2-3	
Leukoencephalopathy	Grade 2-3	
Non-hematological toxicity	Grade 4	
Any adverse events which occurred with dose of 570 mg/m2 of GEM and 44mg/m2 of DTX.		

^a^*G-CSF* Granulocyte colony stimulating factor, *GEM* Gemcitabine, *DTX* Docetaxel

### Outcomes

#### Primary endpoint

The primary endpoint is progression-free survival (PFS), calculated as time from enrolment until first objective documentation of disease progression, treatment failure, or death from any cause.

#### Secondary endpoints

Secondary endpoints are objective tumour response, calculated as overall response rate and disease control rate, along with time to onset of response. Assessment of response and progression is based on RECIST version 1.1 [13]. Overall survival (OS) will be calculated as time from enrolment until death from any cause. Safety and tolerability will be assessed continuously throughout the study. Adverse events will be graded using the National Cancer Institute Common Terminology Criteria for Adverse Events version 4.0 (CTCAE v4.0) [14]. The worst grade of an event during the observation period will be used to denote the severity of that adverse event. Primary and secondary prophylaxis of neutropenia will be routinely assessed and documented in each cycle. Repeated physical examinations will be conducted throughout the study period (consisting of the treatment and post-treatment follow-up periods), including assessment of vital signs and haematological and chemical laboratory tests.

### Statistical analysis

The per protocol set (PPS) will consist of the full analysis of subjects enrolled in this study, excluding those lacking baseline data or with any significant protocol violations involving the study method or concomitant therapy. The safety analysis set will consist of all patients enrolled in this study who received at least one dose of the study drugs. The primary analysis of PFS will be performed on the PPS 1 year after the end of the enrolment period. A confirmatory analysis of all secondary endpoints will be performed at the end of the follow-up period. Kaplan–Meier curves will be used to analyse PFS, OS, and median survival time and 95% confidence intervals for point estimates will be calculated using Greenwood’s formula. The safety endpoint of this study is the frequency of adverse events. No interim analysis is planned.

## Discussion

Bone sarcomas and STS are rare and heterogeneous malignant tumours that account for approximately 1% of all malignant tumours. Osteosarcoma, the most common primary malignant bone tumour, is treated by a standard chemotherapy regimen including high-dose methotrexate, cisplatin, and doxorubicin. Ifosfamide is also an established active agent for osteosarcoma. Ewing sarcoma, a malignant, small round-cell tumour of bone and soft tissue, is treated by multi-agent chemotherapy schedules with vincristine, doxorubicin, and cyclophosphamide (VDC)/ifosfamide and etoposide (IE). Because other malignant primary bone tumours are extremely rare, there is no standard chemotherapy for them and they are treated with regimens similar to those for osteosarcoma. For STS, the mainstream treatment has been various combinations incorporating doxorubicin and ifosfamide. Recently, the agents pazopanib, trabectedin, and eribulin were approved, providing treatment options for second-line or later therapy for patients with STS. However, no optimal therapeutic strategies for histologic subtypes of advanced STS have yet been established.

This study aims to evaluate the efficacy and safety of combination therapy with GD for bone sarcoma and STS. If GD therapy proves to be acceptable, we can provide it as second, third or later lines for sarcoma patients (especially for bone sarcomas). In the future, the role of these treatments, including GD therapy, in specific subtypes of sarcoma may be further clarified.

Limitations of this study include that the sample size is small and that patients with all subtypes of sarcoma will be enrolled, including both bone and soft tissue lesions. A multi-institutional study is needed to achieve a larger sample size and investigate the efficacy of GD therapy for individual subtypes in Japan.

## Additional files

Additional file 1: Data manegement and informed consent procedure. (DOCX 23 kb)

Additional file 2: Procedure for implementation of study monitoring. (DOCX 19 kb)

## Data Availability

The study results, data, and intellectual property rights from this study will belong to the Department of Orthopaedic Surgery of Kobe University Hospital. Specific aspects of handling and distribution will be decided after discussion. Whether the intellectual property will be owned personally by the study representative, principal investigator at the research institution, or the Department of Orthopaedic Surgery will be determined according to the rules of the research institution. Data management and informed consent procedure are indicated in a separate file (Additional file 1). Furthermore, procedure for implementation of this study monitoring is indicated in a separate file (Additional file 2).

## Abbreviations

ALT (GPT): Alanine aminotransferase (glutamyl pyruvic transaminase)
AST (GOT): Aspartate aminotransferase (glutamyl oxaloacetic transaminase)
CTCAE: Common Terminology Criteria for Adverse Events
ECOG: Eastern Cooperative Oncology Group
eGFR: Estimate glomerular filtration rate
GD: Gemcitabine and docetaxel
PFS: Progression-free survival
RECIST: Response Evaluation Criteria in Solid Tumours
STS: Soft tissue sarcoma
