# Supplementary material for: Gemcitabine and docetaxel combination chemotherapy for advanced bone and soft tissue sarcomas: protocol for an open-label, non-randomised, Phase 2 study
Source: BMC Cancer. 2019 Jul 23;19:725. doi: 10.1186/s12885-019-5923-7 (PMC6651911; doi:10.1186/s12885-019-5923-7)
Supplement: Supplementary file 1 — Data manegement and informed consent procedure. (DOCX 23 kb) [file 12885_2019_5923_MOESM1_ESM.docx]

Data management.

Completion of the case report form (CRF)

The principal investigator or sub-investigator will complete the CRF for each subject.

To make a correction to any entry in the CRF, a double line will be struck through the original incorrect data, which should remain legible, and the correct data will be additionally entered. All corrections must be dated and signed (or sealed) by the person making the correction. Except minor corrections (such as correction of a clerical error), all corrections should be accompanied by the reasons.

The principal investigator will confirm that each completed CRF has no incorrect entries, sign the CRF, and then make a copy for filing before submission. The copy of the CRF will be appropriately retained along with the medical record etc.

Specific data in the CRF serving as source document (source data)

In this study, source documents (source data) include the following:

1. Record regarding the subject informed consent and provision of information to the subject
2. Record regarding administration of the study drug
3. Documents or records regarding the studies related to this study required to conform guidelines

Of the data recorded in the CRF, the data in the CRF on the following items will serve as source data. However, if the data are recorded in any other document such as the medical chart, the other document will serve as the source document (source data).

1. Reason for use of the concomitant drug or therapy
2. Adverse event’s severity, outcome (including the outcome at follow-up), seriousness, assessment of the causal relationship to the study drug and the basis for the assessment
3. Reason for discontinuation of the subject from the study
4. Comment of the principal investigator or sub-investigator

Amendment to the protocol and CRF template

To amend the protocol or revise the CRF template, the procedures below should be followed:

1. The principal investigator will promptly submit the amended protocol and the revised CRF template to the head of the research institution, and promptly obtain approval of the ethics review committee via the head of the research institution.
2. When the protocol is amended and the CRF template is revised as instructed by the head of the research institution based on opinions of the ethics review committee within the acceptable range of the study representative and the study coordinating committee, the same procedures will be followed.
3. When the protocol and CRF template need to be changed, the study representative will provide the principal investigators with the draft protocol amendment and the draft CRF template revision, along with the latest package insert and other necessary documents and data.
4. The study representative will give required time to the principal investigators to sufficiently review the provided draft protocol amendment and other documents and data stated above and discuss with the study representative.
5. After discussion with the study representative, the principal investigator will promptly submit the amended protocol and the revised CRF template to the head of the research institution, and promptly obtain approval of the ethics review committee via the head of the research institution.
6. When the protocol is amended and the CRF template is revised as instructed by the head of the research institution based on opinions of the ethics review committee within the acceptable range of the study representative, the same procedures will be followed.

Changes to the statistical analysis plan

If the statistical analysis plan is changed by the responsible biostatistician, the responsible biostatistician will specify all changes in the statistical analysis report of this study. For all changes to the statistical analysis plan, their background will be recorded.

Quality assurance

In this exploratory study performed in a small number of patients, no auditing is planned.

Subject informed consent procedure

Preparation and revision of the informed consent form and other information documents

The principal investigator or sub-investigator will prepare the informed consent form and other information documents to obtain consent to participate in the study from the subjects or their legally acceptable representatives, as well as the informed assent form to obtain assent to participate in the study from the subjects, using simple language as far as possible. The informed consent form, other information documents, and informed assent form will be revised when necessary. The principal investigator or sub-investigator will provide potential subjects with opportunities to ask questions and ample time to decide whether or not to provide consent, and should confirm sufficient understanding of the potential subjects about the contents of this study, before obtaining voluntary consent to participate in this study.

The principal investigator or sub-investigator will submit the prepared or revised informed consent form, other information documents, and informed assent form to the ethics review committee and obtain approval.

No re-consent of subjects will be required for minor changes (e.g., change of the principal investigators at a collaborative research implementing entity) that would not affect the subjects’ will to participate in this study.

Procedure for obtaining informed consent from legally acceptable representative etc.

Given the age distribution of the target disease, this study would be infeasible without enrollment of underage patients. Thus, underage patients will be included in this study. And consent of the subjects’ legally acceptable representatives will also be obtained in this study.

Procedure for obtaining informed assent

The principal investigator or sub-investigator will hand the informed assent form to the potential subjects, and provide sufficient explanation about the contents of the study. The principal investigator or sub-investigator should confirm sufficient understanding of the potential subjects about the contents of this study, and then obtain written assent of the subjects, before initiation of screening examination.

Retention of records, documents etc.

In accordance with the “Guideline on the study data storage period at Kobe University Graduate School of Medicine etc.”, the study representative will retain the study-related important documents (e.g., copies of the application forms submitted to the ethics review committee, notification documents from the head of the research institution, copies of various application forms and reports, documents for data disclosure, and other documents or records supporting the information used in the study including the data correction log and description in notebooks) in a lockable place, either until 10 years after study discontinuation/completion or 10 years after the date of publication of the study results in literature etc., whichever is later. Thereafter, the documents will be destroyed after individuals are made non-identifiable.

The samples (experimental samples or specimens), devices, etc. related to this study will be retained until 5 years (in principle) after the date of the report on last publication of the study results, and thereafter will be destroyed after individuals are made non-identifiable.

At each collaborative research implementing entity, the study data etc. will be appropriately retained for the duration required by the rules of the collaborative research implementing entity.

Compensation for health injury

If a subject suffers health injury related to this study, the sub-investigator will perform appropriate action and treatment. The tests, treatments, and other necessary procedures will be performed within the range of medical services covered by the subject’s health insurance. No financial compensation will be prepared for subjects suffering health injury resulting from this study.
