# Supplementary material for: Gemcitabine and docetaxel combination chemotherapy for advanced bone and soft tissue sarcomas: protocol for an open-label, non-randomised, Phase 2 study
Source: BMC Cancer. 2019 Jul 23;19:725. doi: 10.1186/s12885-019-5923-7 (PMC6651911; doi:10.1186/s12885-019-5923-7)
Supplement: Supplementary file 2 — Procedure for implementation of study monitoring. (DOCX 19 kb) [file 12885_2019_5923_MOESM2_ESM.docx]

Procedure for implementation of study monitoring

- Requirements for study monitors

Study monitors must fulfill the following requirements:

① Understand the ethical principles related to the study

② Have scientific and clinical knowledge needed to perform monitoring activities

③ Continuously receive education, training, etc. about ethical guidelines and applicable laws and regulations

④ Thoroughly familiar with the contents of the study protocol, informed consent form, study-related written procedures, etc.

⑤ Appointed as a study monitor by the principal investigator of this study

⑥ Educated about study monitoring

- Implementation of study monitoring

Monitoring on subject data

During the study, the monitor will have direct access to the source documents etc. (e.g., informed consent form, medical chart, CRF) at Kobe University Hospital for verification. The following items will be checked at the monitoring, using a check list.

a. Informed consent procedure

The monitor will verify that informed consent was obtained appropriately (including that written consent was obtained from the subject in person [or legally acceptable representative etc.] before the start of study treatment). In addition, the monitor will confirm that the informed consent form is appropriately retained.

b. Subject eligibility (Enrollment)

The monitor will verify that the subjects fulfilled the protocol-specified inclusion criteria and did not meet any of the exclusion criteria.

c. Progress of study procedures and CRF completion in individual subjects

The monitor will verify that protocol-specified tests, observations, and assessments were performed according to the study schedule, that the safety of subjects who discontinued study treatment, if any, was appropriately confirmed, and that the data in CRFs are accurate and consistent with source documents.

d. Handling of adverse events

The monitor will check whether any adverse events, including abnormal laboratory values, occurred or not, and all experienced adverse events were properly handled as adverse events in the CRFs. If any serious adverse events occurred, the monitor will also verify that necessary procedures were undertaken (e.g., reporting to the head of the research institution, reporting to the regulatory authorities).

e. Deviations

If any deviations from the protocol or standard operating procedures were noted, the monitor will verify that responses, actions, and preventive measures were taken for the deviations. The monitor will also verify that necessary procedures were undertaken (e.g., reporting to the head of the research institution).

The frequency of study monitoring is as follows:

For “a.” above, the monitoring will be performed every 6 months, on all patients enrolled during the interval.

For “b.” and “c.” above, the monitoring will be performed every 6 months, on one or more patients sampled from all patients enrolled during the interval. The same sampled patients will be subject to the continuous monitoring up to completion of the observation.

For “d.” and “e.” above, the monitoring will be performed every 6 months. All serious adverse events and serious deviations, if any, will be monitored.

The frequency of study monitoring is as follows:

For “a.” above, the monitoring will be performed every 6 months, on all patients enrolled during the interval.

For “b.” and “c.” above, the monitoring will be performed every 6 months, on one patient sampled from all patients enrolled during the interval. The same sampled patient will be subject to the continuous monitoring up to completion of the observation.

For “d.” and “e.” above, the monitoring will be performed every 6 months. All serious adverse events and serious deviations and the patients with the serious adverse events or serious deviations, if any, will be monitored.

The central monitor will perform the study monitoring (i.e., central monitoring) in principle twice a year based on the study data collected at the study secretariat (or the data center) in terms of the following items. However, if judged to be necessary by the study representative or the principal investigator, on-site monitoring (including source data verification) will be performed.

① Subject enrollment status

② Subject eligibility at enrollment

③ CRF submission status / EDC data entry status

④ Missing data and outliers

⑤ Presence or absence of adverse events

⑥ Protocol deviations

⑦ Non-compliance with the protocol etc.

⑧ Other variables affecting implementation of the study

Only regarding informed consent, the central monitor will check the following with the principal investigator:

· That informed consent was obtained appropriately (including that written consent was obtained from the subject in person [or legally acceptable representative] before the start of study treatment)

· That the informed consent form is appropriately retained.

Monitoring on non-subject data

The monitor will perform study monitoring using a check list at the time points specified below. However, study monitoring before the start of the study will be performed only after approval of the ethics committee, that during the study will be performed twice a year (in principle), and that after the end (discontinuation or termination) of the study will be performed at submission of the study closure report.

１) Before the start of the study

① Ethics review committee approval status

The monitor will verify that approval of the ethics review committee was obtained for all necessary procedures before the start of the study.

② Registration with a public study registry database

The monitor will verify that this study has been registered with a clinical study registry database.

③ Conflicts of interest

The monitor will verify that all potential conflicts of interest of investigators were reviewed by the ethics review committee etc.

④ Study organization requirements

The monitor will verify the standard operating procedures of Kobe University Hospital. In addition, the monitor will verify a list of the investigators involved in this study, and that these investigators received education/training.

⑤ Document preparation and retention status

The monitor will verify that the documents required by the “Ethical Guidelines for Medical and Health Research involving Human Subjects”, “Ethical Guidelines for Human Genome and Gene Analysis Research (if applicable)”, standard operating procedures, etc. were prepared and are appropriately retained.

２) During the study

３) After the end of the study (discontinuation or termination)

- Monitoring report

Preparation of the monitoring report

The monitor will prepare a monitoring report describing the following:

(1) Date, time, and location of the monitoring

(2) Monitor’s name and affiliation

(3) Summary of monitoring results

Submission of a monitoring report

The monitor for each research institution will submit a monitoring report to the principal investigator of the research institution, study secretariat, and central monitor after implementation of monitoring. The central monitor will submit a monitoring report to the study representative and the principal investigators of collaborative research implementing entities after implementation of monitoring.

- Confidentiality obligation

Study monitors must not leak any information learned in the course of the work without due reasons, even after leaving the work.

- Retention of documents, etc.

The principal investigator will retain this written procedure and the forms and documents related to the procedures specified in this document, in a cabinet for document storage under lock and key, for the period specified in the protocol.
